# A Case of Self-Immolation in a Woman With Recurrent Puerperal Psychosis From Pakistan

**DOI:** 10.1192/bjo.2024.651

**Published:** 2024-08-01

**Authors:** Muhammad Usman Amjad, Anum Zahra

**Affiliations:** 1 Children's Health Ireland (CHI), Dublin, Ireland; 2 DHQ Teaching Hospital and Medical College, Dera Ghazi Khan, Pakistan

## Abstract

**Aims:**

Postpartum psychiatric disorders are almost certainly common among women in Pakistan but accurate estimates of the prevalence of these disorders are difficult to obtain because of cultural norms and lack of awareness that may result in women underreporting such disorders, or them not being recognised because of lack of reliable screening tools and resources.

The aims of this case study are to report a case of an attempted suicide by self-immolation in a multiparous woman with recurrent puerperal psychosis, highlighting the cultural/religious barriers which often result in delayed help, and call attention to the need for awareness and screening.

**Methods:**

A 35-year-old multiparous woman, hailing from low socioeconomic background in the outskirts of Dera Ghazi Khan, was admitted to the burns unit of our hospital after setting herself on fire. Psychiatric consultation was sought after obtaining a detailed history from the family members. She had given birth to her fifth child (2^nd^ son) two weeks previously via spontaneous vaginal delivery (SVD). Soon afterwards, she developed low mood and was crying all of the time. She also developed feelings of excessive guilt and worthlessness and started praying excessively and asking for forgiveness of others. At times, she talked about wanting to end her life because she thought she was worthless, sinful, and didn't deserve to live. She also wouldn't come close to her newborn, care for him or even touch him. Her family members had also observed her talking to herself when she was alone.

According to her brother, she had a previous episode shortly after giving birth to her fourth child (1^st^ son) 2 years before. The family believed at first that it was a result of an “evil eye” because she had “finally” given birth to a son after giving birth to three daughters in a row. The patient's mother took her to a faith healer which did not result in any improvement. When her condition deteriorated, they took her to a psychiatrist in their hometown who started her on psychotropic medication. Her condition improved after a few months.

After this baby her symptoms were reported to be much more severe with active suicidal ideation. Her family members couldn't take her to that same doctor because he had moved to another city. Also her previous prescriptions were lost so they had no record of the medication the patient had been on before. In addition the patient's mother was totally against the idea of taking her to a medical doctor and was determined to take her to faith healers instead, which further contributed to the delay in her getting medical help. Two weeks after giving birth to her second son she locked herself in her bedroom and set herself on fire. Her family members rescued her and took her to ER. She sustained injuries to her neck, chest, and arms. A diagnosis of puerperal psychosis was made taking into account her history and her mental state examination. She was started on psychotropic medication along with analgesics and antibiotics.

**Results:**

No matter where a woman lives, postpartum psychiatric disorders are a serious issue that can negatively impact a woman's quality of life and well-being if not addressed and treated properly. While these disorders receive adequate attention in developed countries, it is a largely neglected issue in Pakistan, but one that deserves our attention. It can have serious implications if proper medical help is not sought early like in this case. It is, therefore, recommended that all pregnant women who present to their GPs/obstetricians/midwives for antenatal checks should be screened for perinatal psychiatric disorders with a validated instrument and educated accordingly.

**Conclusion:**

As this patient had a previous episode of puerperal psychosis, she was at a very high risk of this relapse and it could have been prevented, or treated early after the birth if this fact was widely known and recognised.

(A photograph of the patient's burn wounds taken after skin grafting will be added to the poster once the abstract is approved. No financial sponsorship. The work was conducted with appropriate ethical and governance safeguards, which also include obtaining family's consent.)

